# In situ sequence-specific visualization of single methylated cytosine on tissue sections using ICON probe and rolling-circle amplification

**DOI:** 10.1007/s00418-022-02165-2

**Published:** 2022-11-23

**Authors:** Sohei Kitazawa, Ryuma Haraguchi, Yuki Takaoka, Riko Kitazawa

**Affiliations:** 1 Department of Molecular Pathology, Ehime University Graduate School of Medicine, Shitsukawa 454, Toon, Ehime 791-0295 Japan; 2 Division of Diagnostic Pathology, Ehime University Hospital, Shitsukawa 454, Toon, Ehime 791-0295 Japan

**Keywords:** DNA methylation, In situ hybridization, ICON probe, Padlock probe, Rolling-circle amplification, Mitochondria DNA, FFPE

## Abstract

Since epigenetic modifications differ from cell to cell, detecting the DNA methylation status of individual cells is requisite. Therefore, it is important to conduct “morphology-based epigenetics research”, in which the sequence-specific DNA methylation status is observed while maintaining tissue architecture. Here we demonstrate a novel histochemical technique that efficiently shows the presence of a single methylated cytosine in a sequence-dependent manner by applying ICON (interstrand complexation with osmium for nucleic acids) probes. By optimizing the concentration and duration of potassium osmate treatment, ICON probes selectively hybridize to methylated cytosine on tissue sections. Since the elongation process by rolling-circle amplification through the padlock probe and synchronous amplification by the hyperbranching reaction at a constant temperature efficiently amplifies the reaction, it is possible to specifically detect the presence of a single methylated cytosine. Since the ICON probe is cross-linked to the nuclear or mitochondrial DNA of the target cell, subsequent elongation and multiplication reactions proceed like a tree growing in soil with its roots firmly planted, thus facilitating the demonstration of methylated cytosine in situ. Using this novel ICON-mediated histochemical method, detection of the methylation of DNA in the regulatory region of the RANK gene in cultured cells and of mitochondrial DNA in paraffin sections of mouse cerebellar tissue was achievable. This combined ICON and rolling-circle amplification method is the first that shows evidence of the presence of a single methylated cytosine in a sequence-specific manner in paraffin sections, and is foreseen as applicable to a wide range of epigenetic studies.

**Supplementary Information:**

The online version contains supplementary material available at 10.1007/s00418-022-02165-2.

## Introduction

Epigenetics is a mechanism that regulates gene expression patterns by changing genome structure and function in response to the environment (Seton-Rogers 2015; Waddington 1959; Fujii et al. 2021; Sato and Sassone-Corsi 2022; Cedar et al. 2022). It is becoming clear that this mechanism is not only closely related to cell differentiation, nuclear reprogramming, and aging signals, but also to several disease processes such as cancer (Skourti and Dhillon 2022; Issa 2000; Toyota and Issa 2000; Esteller 2007), diabetes (Mahajan et al. 2022; Ling et al. 2022), atherosclerosis (Lund and Zaina 2007; Turunen et al. 2009), and lifestyle-related conditions (Riancho 2015). Epigenetics is ultimately interpreted as a control mechanism of gene expression by changing the chromatin structure (Ordog et al. 2012). Three typical epigenetic control mechanisms affect such regulation: (1) histone protein modification (Cedar and Bergman 2009; Zhang et al. 2021), (2) non-coding RNA (Kleaveland et al. 2018; Wei et al. 2017) and (3) DNA methylation (Cedar et al. 2022). The interaction among these three mechanisms aggregates or relaxes the chromatin structure to reduce, stop, enhance, or start gene expression (Goldberg et al. 2007). Among these three mechanisms, DNA methylation is regarded as a major epigenetic landmark (Meier and Recillas-Targa 2017). Indeed, when cells divide and DNA is replicated, the methylated or unmethylated state is immediately maintained by DNA methyltransferase as cell memory (Jones and Liang 2009; Petryk et al. 2021). This maintenance methylation process is, however, more unstable than conventional nucleotide duplication at cell division, and therefore facilitates the epigenetic diversity of cell types from a single fertilized egg (Petryk et al. 2021).

Histochemical techniques have been developed to demonstrate epigenetic alterations at tissue or cell level. Various specific antibodies against modified histone proteins have been developed (Hayashi-Takanaka et al. 2020), and are already being applied to the histopathological diagnosis of giant cell tumors of bone (Behjati et al. 2013; Ishihara et al. 2022), chondroblastoma (Cleven et al. 2015) and some brain tumors (El-Hashash 2021). For the DNA methylation status, immunohistochemistry using an antibody that recognizes methylated cytosine has been developed to demonstrate the presence of methylated cytosine (Frediani et al. 1986). Nonetheless, the antibody simply provides a ubiquitous nuclear staining pattern and, therefore, the biological information obtained is limited. The method using microdissection is also available (Kitazawa et al. 2018), but since the DNA methylation status may differ between neighboring cells, it requires isolation of single cells from tissues, which limits epigenome analysis with the use of this method.

Here, we demonstrate a novel histochemical technique that efficiently displays the presence of a single methylated cytosine in a sequence-dependent manner by applying an ICON (interstrand complexation with osmium for nucleic acids) probe (Okamoto and Tainaka 2005).

## Materials and methods

### Designing ICON, padlock, and branching probes (Fig. 1)

**Fig. 1:**
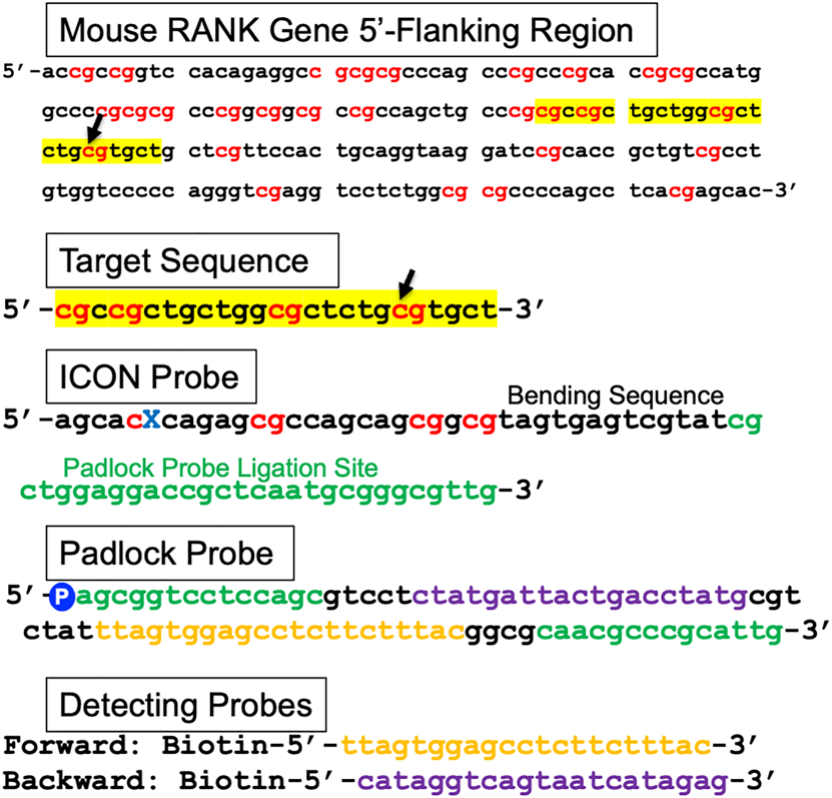
Designing ICON, padlock, and branching probes. The objective sequence is located 5'-upstream of the murine RANK gene (*upper panel*). This region contains numerous CpG sites (*cg in red*) forming typical CpG islands. The target sequence gcgccgctgctggcgctctgcgtgct (*yellow highlighted part*) was set to detect the methylation of cytosine (*arrows*). The ICON probe, 5-agc acX cag agc gcc agc agc ggc gta gtg agt cgt atc gct gga gga ccg ctc aat gcg ggc gtt g-3, is designed complementary to the target sequence of murine RANK gene (*underlined*), where X is the modified nucleotide to cross-link to the corresponding methylated cytosine. The customized ICON probes were synthesized at GeneDesign Co. Ltd. (Ajinomoto Bio-Pharma Services, Tokyo, Japan, https://www.ajioligos.com/en/products/nucleic-acid-medicine-oligos/). The ICON probe has a bending sequence followed by the padlock probe ligation site (*green*) to which the padlock probe binds during the subsequent rolling-circle amplification reaction. The sequence of the padlock probe is set to be placed at both ends with a free phosphoric acid group at its 5’-end to form a circular structure when hybridized to the padlock probe ligation site of the ICON probe. The primers used for the hyperbranching amplification reaction were prepared by synthesis of biotin-labeled 5'-side primers for both forward (F) and branching (B). F: ttagtggagcctcttctttac (*yellow*), B: cataggtcagtaatcatagag (*purple*) are synthesized and used during the hyperbranching reaction. Each primer is designed to bind to each colored part of the padlock probe. The negative control is prepared by replacing the target-specific sequence of each ICON probe with a shuffled non-specific one

The objective sequence selected is located in the 5'-upstream region of the murine receptor for the osteoclast differentiation factor, the receptor activator of nuclear factor κB (RANK, also known as the TRANCE receptor, TNFRSF11A) gene (Ishii et al. 2008). This region contains numerous CpG sites (red cg in Fig. 1) forming typical CpG islands (part of the sequence is as follows: cg sites are in bold face; 5-ac**c g**cc ggt cca cag agg c**cg cgc g**cc cag cc**c g**cc **cg**c ac**c gcg** cca tgg ccc **cgc gcg** cc**c g**g**c g**g**c g**c**c g**cc agc tgc c**cg cgc cgc tgc tgg cgc tct gcg tgc t**gc t**cg** ttc cac tgc agg taa gga tc**c g**ca c**cg** ctg t**cg** cct gtg gtc ccc cag ggt **cg**a ggt cct ctg g**cg cg**c ccc agc ctc acg agc acc agg tga aac t**cg** ggg tat c**cg** gat gga gac ccc agc tgg-3). From these CpG islands, the ICON probe was designed to hybridize to gcgccgctgctggcgctctgcgtgct (underlined in the above sequence, yellow high-lighted part in Fig. 1) and to detect the methylation of cytosine (arrows, Fig. 1). The designed ICON probe has the following sequence: 5-agc acX cag agc gcc agc agc ggc gta gtg agt cgt atc gct gga gga ccg ctc aat gcg ggc gtt g-3. The underlined part is the complementary sequence that binds to part of the murine RANK gene, and the X is a modified nucleotide to cross-link to the corresponding methylated cytosine. The customized ICON probes were synthesized at GeneDesign Co. Ltd. (Ajinomoto Bio-Pharma Services, Tokyo, Japan (https://www.ajioligos.com/en/products/nucleic-acid-medicine-oligos/)). The sequence shown in green in Fig. 1 is the one to which the padlock probe (Landegren et al. 1996) binds during the subsequent rolling-circle amplification reaction (Zhang et al. 1998; Watanabe et al. 2011). This sequence can be designed arbitrarily as long as there is no homology to the DNA sequence of the organism to be examined. By changing the individual padlock probe ligation sequence of the ICON probe, multicolored staining is possible in the subsequent amplification reaction (supplementary Fig. 1). The sequence of the padlock probe is set to be placed at both ends with a free phosphoric acid group at its 5’-end to form a circular structure when hybridized to the padlock probe ligation site of the ICON probe. Using the circular form of the padlock probe as a template, the tail part on the 3'-side of the ICON probe then expands while repeating the same base sequence just like a “roller-type stamp” (Watanabe et al. 2011). The primers used for the hyperbranching amplification reaction were prepared by the synthesis of biotin-labeled 5'-side primers for both forward (F) and branching (B) primers. F: ttagtggagcctcttctttac (yellow, Fig. 1) and B: cataggtcagtaatcatagag (purple, Fig. 1) are synthesized and used during the hyperbranching reaction. Each primer is designed to bind to each colored part (in Fig. 1) of the padlock probe. The same primer combination is used repeatedly for each padlock probe sequence. Fluorescent labeling is achieved by using various fluorescent labeling primers instead of biotin labeling (Alexa Fluor 488 for green and Alexa Fluor 594 for red). All customized oligo DNA were purchased from ThermoFisher Scientific. Similarly, the ICON probe for detecting methylated cytosine at murine mitochondrial DNA D-loop (Stoccoro et al. 2022; Bianchessi et al. 2016) was designed as follows: 5- cgtatgggcXataacgcatttgatggccctgtagtgagtcgtatcgctggaggaccgctcaatgcgggcgttg-3 (the underlined part is specific to the mitochondrial DNA sequence). The negative control is prepared by replacing the target-specific sequence of each ICON probe with a shuffled non-specific one.

### Specificity of ICON probe binding to the target methylated sequence on nylon membrane (Fig. 2a)

**Fig. 2:**
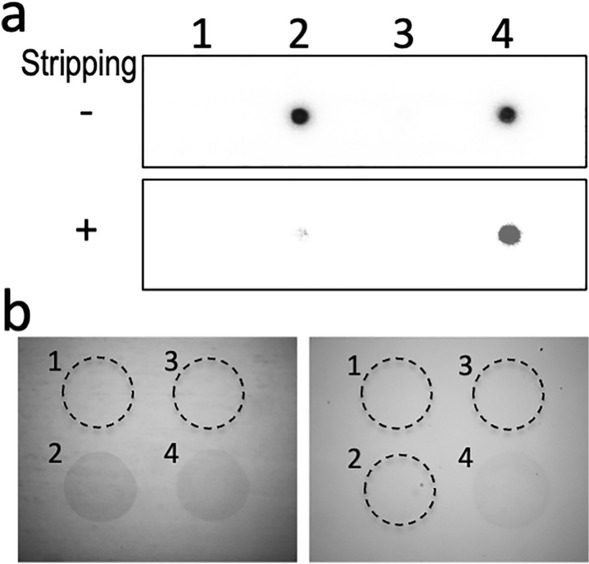
The ICON probe differentially detects single methylated cytosine in a sequence-dependent manner. Dot-blot hybridization (**a**) demonstrates that without stripping the membrane, the radiolabeled oligo DNA probe hybridizes both the unmethylated and methylated plasmids with target DNAs (*lanes 2 and 4*). After stripping, while the un-crosslinked probe was stripped off by boiling, the hybridized probe crosslinked by potassium osmate treatment remained in the methylated plasmid with target DNA (*lane 4*). On the glass slides (**b**), conventional hybridization followed by potassium osmate reaction, and washing without denaturing by NaOH, the ICON probe binds to both the methylated and unmethylated target DNA (**b**,* left*, Nos. 2 and 4). On the other hand, after washing with NaOH and removing the un-crosslinked probe, only the ICON probe remained in the methylated target on glass slides (**b**, No. 4)

The 5’-flanking region of the mouse RANK gene was cloned as described (Ishii et al. 2008), and the 1 kb upstream from the transcription start site was ligated onto a pGL3 Basic vector (Promega, Madison, WI, USA) yielding a pGL3-RANK-1. The plasmid vector with or without the RANK gene promoter region was treated with SssI methylase (New England Biolabs, Ipswich, MA, USA, cat# M0226S) to yield a construct with methylation at all CpG sites. One pg of four types of plasmid constructs: (1) vector without methylation, (2) vector + RANK gene promoter without methylation, (3) vector with methylation, and (4) vector + RANK gene promoter with methylation, was spotted onto the Hybond N + nylon membrane (Amersham Biosciences Corp., Piscataway, NJ, USA) and immobilized by UV cross-linking. The blotted membrane was prehybridized with prehybridization buffer in a sealed bag at 50 °C in a water bath for 2 h. The prehybridization buffer was replaced with hybridization solution (hybridization buffer containing 100 ng/ml of ICON probe for RANK gene promoter end-labeled with γP^32^-ATP with T4 polynucleotide kinase (Takara, Tokyo, Japan) to a specific activity of 2 × 10^8^ cpm/ µg. The membranes were hybridized at 50 °C for 2 h, washed in 2 × SSP containing 0.1% SDS, and in 1 × SSP containing 0.1% SDS, then in a 0.1 × SSP containing 0.1% SDS. For the potassium osmate reaction, the washed membrane was treated with freshly prepared K_3_Fe(CN)_6_ (Wako, Tokyo, Japan) in 50 mM Tris–Cl, pH 7.7, 0.5 mM EDTA, 1 M sodium chloride at 55 °C for 10 min, and then with a freshly prepared potassium osmate solution containing 0.1 M K_3_Fe(CN)_6_, 5 mM K_2_OsO_4_ (Sigma-Aldrich, Japan) in 50 mM Tris–Cl, pH 7.7, 0.5 mM EDTA, 1 M sodium chloride at 55 °C for 1 h basically, according to the protocol described by Tainaka and Okamoto (2011). After cross-linking by potassium osmate, the membrane was rinsed three times with 0.1 × SSP containing 0.1% SDS at 37 °C, then analyzed with image analyzer BAS2000 (FUJIX, Tokyo, Japan) to confirm the conventional dot-blot hybridization reaction. After the first imaging, the membrane was treated for 5 min with boiling H_2_O containing 0.1% SDS for stripping the hybridized probe, and reanalyzed with BAS2000 imaging.

### Optimizing specificity of ICON probe binding to target methylated sequence on glass slide (Fig. 2b)

Prior to the histochemical application of the ICON probe, the reaction was optimized for the non-isotopic demonstration of spotted oligonucleotide with or without single methylated cytosine on glass slides. Part of the RANK gene promoter region (tct ggt tct tac ttc agg gcc atc aaa tgc tgg atc* gcc cat acg) and its shuffled sequence (gca agt cct aac gtc tgt gtt atc tga ggc gtc tac* gaa ctc cgc) were synthesized with 5’-amino modification (Hokkaido System Science, Co., Ltd., Sapporo, Japan) with or without methylation at * site, and with 0.1 pg each of four types of oligonucleotide: (1) shuffled RANK without methylation, (2) RANK without methylation, (3) shuffled RANK with methylation, and (4) RANK with methylation, spotted onto a SDA0011 microarray of glass slides (Matsunami Glass Ltd, Osaka, Japan) for covalent immobilization of amino-modified oligoDNA by using the VP478A DNA manual arrayer (V & P Scientific, CA, USA). The slides were then kept overnight in a humidified box with saturated NaCl solution at 37 °C. After soaking in distilled water, the spotted slides were air-dried and stored in a desiccator until use. To optimize specificity and sensitivity of in situ non-radioactive detection of methylated cytosine on glass slides, a series of conditions (concentration and duration of K_2_OsO_4_ treatment with or without K_3_Fe(CN)_6_ solution for cross-linking, and stripping solutions at various temperatures) were tested.

### Padlock ligation, hyperbranching-rolling-circle amplification (H-RCA) reaction

The composition of the padlock ligation reaction solution was adjusted to a total volume of 50 µl (padlock probe (1 pmol/µl) 5 µl, 10 × Taq-DNA buffer 5 µl, Taq DNA ligase (New England Biolabs, cat# M0208S) 2.5 µl, sterilized water 37.5 µl). The reaction solution was then layered on the section, treated at 94 °C for 10 min, and allowed to stand in a closed container preheated at 60 °C for 60 min to complete the ligation reaction. After washing three times with PBS for 5 min at 37 °C, the specimen was washed thoroughly by dipping the slide several times in distilled water. The composition of the H-RCA reaction solution was adjusted to 50 µl: (forward primer (10 pmol/µl) 1 µl, backward primer (10 pmol/µl) 1 µl, 2 × Reaction Mix 25 µl, Bst DNA polymerase (Eiken Chemical Co., Ltd., Tokyo, Japan, cat# LMP204) 2 µl, sterilized water 21 µl). The detection primers used for the amplification reaction were prepared by customized synthesis with 5’-biotin-labeled (Fig. 1) to incorporate biotin into the amplified DNA together with the hyperbranching reaction. The reaction solution was layered on the section and allowed to stand for 30 min in a closed container preheated at 65 °C to complete the amplification reaction. After washing three times in PBS for 5 min at 37 °C, the specimen was thoroughly washed by dipping the slide several times in distilled water. Strept-Avidin-HRP complex (Bio-Techne, MN, USA, cat# 4800-30-06) diluted 50-fold with PBS was added to cover the specimen in a closed container preheated at 37 °C for 10 min. After washing 3 times with PBS by gentle shaking for 5 min at 37 °C, the colorimetric reaction was completed by adding DAB under a microscope until a round dot-like spot was observed (usually within 3–5 min). The specimen was then washed with water for 1 min to stop color development, and again briefly washed with water. The specimen was then dehydrated with series of 70, 80, 90, and 100% graded alcohol, and finally immersed in xylene and covered with a glass cover.

### Cell and tissue preparation (Fig. 3)

**Fig. 3:**
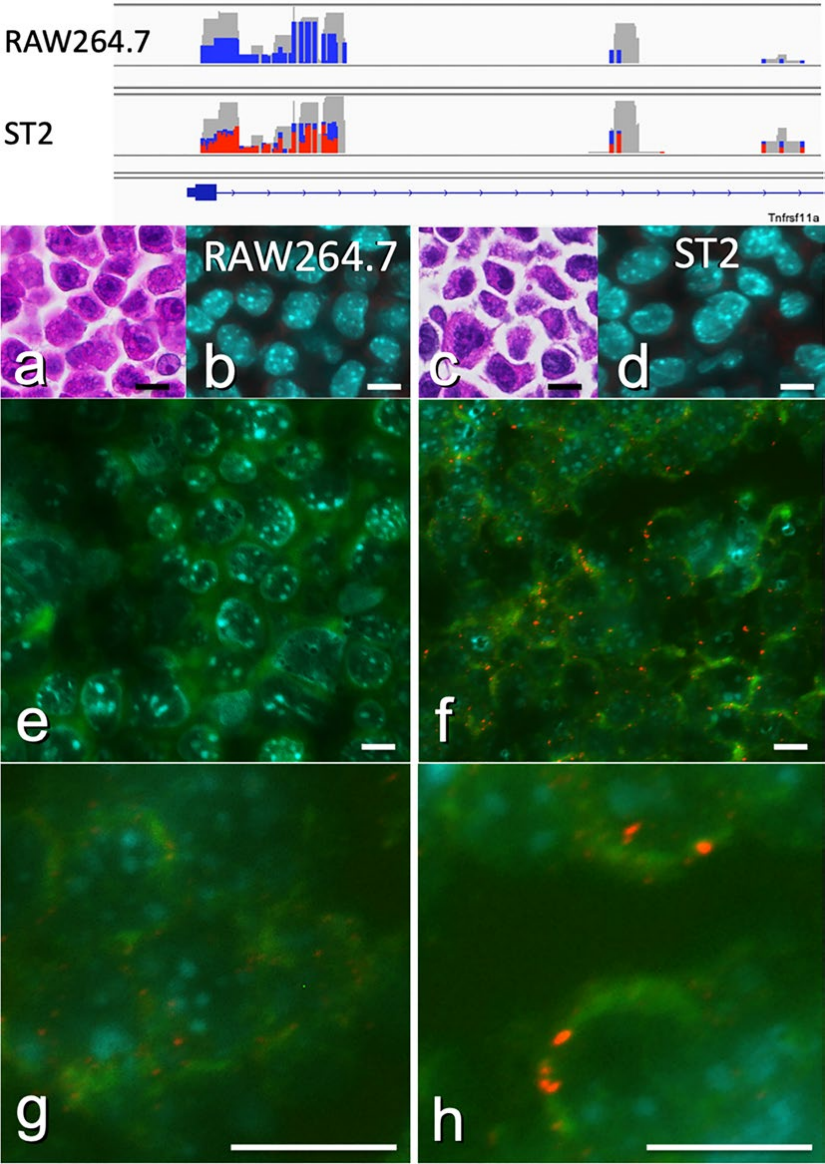
Identification of RANK gene promoter methylation by ICON histochemistry. By epigenomic analyses (*upper panel*), the CpG island located at the 5’ transcription start site is hypomethylated (*blue*) in RAW264.7 cells and hypermethylated (*red*) in ST2 cells. Because ICON histochemistry requires, unlike conventional fluorescent ISH (FISH) study, repeated denature, hybridization and development process, preservation of cell structure is impaired at higher magnification as shown in** g** and** h**. In spite of this drawbacks, histochemical demonstration targeting these two cell lines (**a** and** b**, HE) shows that ICON histochemistry differentiates these two cells; while red signals by Alexa Fluor 594 are scarcely observed among CpG-hypomethylated RAW264.7 cells (**e** and** g**), those of CpG-hypermethylated ST2 cells are seen, mostly as one or two red spots at the marginal area in the nuclei (**f** and** h**). Negative controls prepared by shuffled sequence do not show any significant signals (**b** and** d**). Each* scale bar* indicates 20 µm

With the use of optimized hybridization, washing and denaturing conditions on glass slides, as described in the above-mentioned studies, the study was extended to detecting single methylated cytosine at cell and tissue levels. Basically, the following protocol was used for cell and tissue studies.

RAW264.7 cells (RIKEN, Tsukuba, Japan) were cultured and maintained in α-MEM (Sigma-Aldrich), supplemented with 10% FBS (Sigma-Aldrich), 50 I.U./ml-50 µg/ml penicillin/ streptomycin (ICN Biomedicals Inc., Aurora, OH, USA) and 2 mM of l-glutamine (ICN Biomedicals Inc.). Cells of mouse bone marrow stromal cell line ST2 (Riken, Tsukuba, Japan) were cultured in phenol red-free α-MEM (Sigma-Aldrich) supplemented with 2% charcoal-stripped fetal bovine serum, collected by centrifugation (1500 rpm, 5 min) and fixed in 4% paraformaldehyde (PFA)/PBS for 2 h at 4 °C. At the same time, DNA samples were purified and subjected to epigenome analyses (Bisulfite Sequencing Services, Active Motif, Carlsbad, CA, USA). Brain tissues from 16-week-old mice, under anesthesia, were dissected and maintained in 2.5% isoflurane and fixed in 4% PFA/PBS for 2 days at 4 °C, dehydrated through ethanol, and embedded in paraffin; 4-µm serial sections were then prepared for histological analysis. DNA was purified from part of the brain tissue samples, and subjected to bisulfite conversion. After PCR amplification of the mitochondrial D-loop with a set of primers (sense: 5’-GGTTTTTATTTTAGGGTTATTAAATG-3’ and antisense: 5’-CCAAATACATAACACCACAATTATATTAATC-3’), PCR products (121 bps) were ligated to TA-vector (Promega); 12 independent colonies were then selected and sequenced. Mouse brains were dissected, and the tissue fixed in 4% PFA/PBS for 2 days at 4 °C was dehydrated through ethanol and embedded in paraffin; 4-μm serial sections were then prepared for histological analysis. Routine hematoxylin and eosin (HE) staining and immunohistochemical analysis with the use of the primary anti-MTCO1 antibodies (1:1000, cat. no. ab203912, Abcam Plc.) were carried out. Signal detection was carried out with 3,3'-diaminobenzidine (DAB, cat. no. K3468, Dako North America Inc.). All animal experimental procedures and protocols were approved by the Committee on Animal Research at Kobe and Ehime Universities (Permit No. 05-KU-36–16, 9 July 2018).

- (1) Tissue sample deparaffinization, hydrophilization, and pretreatment.
  - (1) After deparaffinizing with xylene and alcohol hydrophilic series, the specimen is immersed in distilled water.
  - (2) Microwave treatment in 0.01 M citric acid buffer (pH 6.0) at 97 °C for 20 min; let it stand and gradually cool to 23 °C to loosen methylene bridge generated by the fixative.
  - (3) Proteinase treatment with pepsin solution (0.3% pepsin/0.01 N HCl, pre-warmed to 37 °C) in a water bath at 37 °C for 10 min. After the treatment, wash the specimen with distilled water twice for 3 min each, then dip several times in PBS solution.
  - (4) Place the 4% PFA solution on slides, and let them stand for 10 min at 23 °C for refixation. Next, wash again three times with PBS for 3 min to thoroughly wash off the PFA fixative. Then dip the slides in distilled water.
  - (5) Dip the specimen in 0.1% Tween20/2 × SSC, pre-warmed at 37 °C, for 30 min to induce aging.
  - (6) After washing with distilled water, dip the specimen in dehydration series (70, 80, 90, and 100%) of ethanol solutions ten times, and finally air-dry the specimen.
  - (7) Layer 70% formamide/2 × SSC preheated at 75 °C on the sample and place it in a humid incubator at 75 °C for 5 min to heat and denature the nucleic acid in the tissue. At the same time, treat the hybridization buffer containing the ICON probe at 75 °C for 5 min and keep it warm at 42 °C.
  - (8) After washing with distilled water, the specimen is dipped in an alcohol dehydration series (70, 80, 90, 100% ethanol solution) ten times and finally air dried.
- (2) Hybridization and conventional washing
  - (1) Add the ICON probe to the hybridization buffer (RNAscope blank probe dilutant-C1, 300041, Advanced Cell Diagnostics, Inc., CA, USA) to a final concentration of 1 pmol/µl. After covering the hybridization solution with a cover slip to prevent evaporation, place it in a closed humified container at 42 °C overnight.
  - (2) After incubation, remove the cover slip, wash the specimen first, with 2 × SSC, 0.5% SDS, for 5 min three times at 23 °C, second, with 0.2 × SSC, 0.1% SDS for 5 min two times at 42 °C, third with 0.2 × SSC, 0.1% SDS, at 23 °C for 5 min, and finally with 0.2 × SSC at 23 °C while dipping the slide in the solution about 5–10 times.
- (3) Osmium reaction and washing to strip off un-crosslinked ICON probe
  - (1) For the potassium osmate solution, adjust the buffer (50 mM Tris–HCl, 1 M NaCl, 0.5 mM EDTA) by excluding potassium osmate from the stock at the time of use, and adjust the final concentration of potassium osmate to 1 mM immediately before use.
  - (2) Overlay the washed specimen with the buffer, excluding potassium osmate, for 2 min at 23 °C. Replace the overlayed buffer with the buffer with potassium osmate, and keep at 37 °C for 15 min. Note that this operating temperature and duration may need minor adjustment according to the fixative condition of the specimen.
  - (3) Remove the potassium osmate and wash the specimen with 0.5 N NaOH solution at 23 °C for 20 min to dissociate the ICON probe, for which a strong bond was not obtained with potassium osmate, from the double-stranded state.
  - (4) Remove the 0.5 N NaOH solution and wash the specimen with 60% DMSO. This DMSO treatment prevents the detached ICON probe from rehybridization.
  - (5) Repeat washing the specimen with PBS for 5 min at 23 °C. Since specimens treated with 0.5 N NaOH are easily peeled off, this PBS washing needs to be done carefully.
  - (6) After soaking in distilled water, the specimen is thoroughly air-dried.
- (4) Padlock ligation and H-RCA reaction
  - (1) Adjust the composition of the Padlock Ligation reaction solution to a total volume of 50 µl (Padlock probe, 1 pmol/µl, 5 µl, 10 × Taq-DNA Buffer 5 µl, Taq DNA ligase 2.5 µl, sterilized water 37.5 µl) according to the manufacturer’s protocol. The reaction solution is layered on the section, treated at 94 °C for 10 min, and then allowed to stand in a closed container preheated at 60 °C for 60 min to complete the ligation reaction.
  - (2) Repeat washing with PBS for 5 min at 23 °C three times, and then replace the PBS with distilled water. Wash thoroughly by dipping the slide several times in the solution and shaking it.
  - (3) Adjust the composition of the H-RCA reaction solution (primer F (10 pmol/µl) 1 µl, primer B (10 pmol/µl) 1 µl, 2 × reaction mix 25 µl, Bst DNA polymerase (Eiken Chemical Co., cat # LMP204) 2 µl, sterilized water 21 µl to a total volume of 50 µl. The reaction solution is layered onto the section and allowed to stand for 30 min in a closed container preheated at 65 °C to complete the amplification reaction.
  - (4) Repeat PBS washing for 5 min × 3 times at 23 °C, and then replace PBS with distilled water. Wash thoroughly by dipping the slide several times in the solution and shaking it.
  - (5) Add strept-avidin-HRP (TREVIGEN, cat# 4800-30-06) diluted 50-fold with PBS to the specimen and let react for 10 min in a closed container preheated at 37 °C.
  - (6) Repeat washing the specimen thoroughly with the PBS three times, while shaking, for 5 min at 23 °C.
  - (7) Observe color development of the DAB under a microscope. Usually, a round dot-like color appears in about 3–5 min. Wash the specimen with water to stop color development, stain with a light green stain for 1 min, and then lightly wash with water. After the alcohol dehydration series, replace with xylene and seal the specimen with a glass cover.

### Image acquisition

All the sections were photographed under a Nikon Eclipse Ci microscope (Nikon, Japan), equipped with a 5.9-megapixel Nikon DS-Fi3 digital camera (Nikon, Japan) using Nikon NIS-Elements D software version 5.11 (Nikon, Japan).

## Results

### ICON probe differentially detects single methylated cytosine in a sequence-dependent manner (Fig. 2)

As shown in Fig. 2a, dot-blot hybridization demonstrated that without stripping the membrane, the radiolabeled oligo DNA probe hybridized both unmethylated and methylated plasmid with target DNAs (Fig. 2a, lanes 2 and 4). After stripping, while the uncross-linked probe was stripped off by boiling, the hybridized probe cross-linked with potassium osmate treatment remained linked to the methylated plasmid with target DNA (Fig. 2a, lane 4). On the glass slides, conventional hybridization followed by potassium osmate reaction, and washing without denaturing by NaOH, the ICON probe bound to both methylated and unmethylated target DNA (Fig. 2b, left, No. 2 and 4). On the other hand, since the potassium osmate reaction was used as for radioactive probes resulted in low selectivity for the methylated target after the NaOH stripping reaction, the K_2_OsO_4_ concentration was serially reduced with and without the K_3_Fe(CN)_6_ solution. The optimal condition for selective demonstration of the methylated target on the glass slide was achieved by adding 1 mM of K_2_OsO_4_ without K_3_Fe(CN)_6_ solution (Fig. 1b, right). This optimized condition was used for the following histochemical applications.

### Identification of RANK gene promoter methylation by ICON histochemistry (Fig. 3)

By epigenomic analyses (Fig. 3 upper panel), the CpG island located at 5’ transcription start site was hypomethylated (blue) in RAW264.7 cells, constitutively expressing the RANK gene (Fig. 3a, HE), and hypermethylated in ST2 cells (Fig. 3c, HE). Histochemical demonstration targeting these two cell lines showed that ICON histochemistry clearly differentiated these two cells, while red signals by Alexa Fluor 594 were scarcely observed among CpG-hypomethylated RAW264.7 cells (Fig. 3e and g); those of CpG-hypermethylated ST2 cells were clearly seen mostly at the marginal area in the nuclei (Fig. 3f and h). Negative controls prepared by shuffled sequence did not show any significant signals (Fig. 3b and d).

### Demonstration of methylated cytosine in mitochondrial DNA (Fig. 4)

**Fig. 4:**
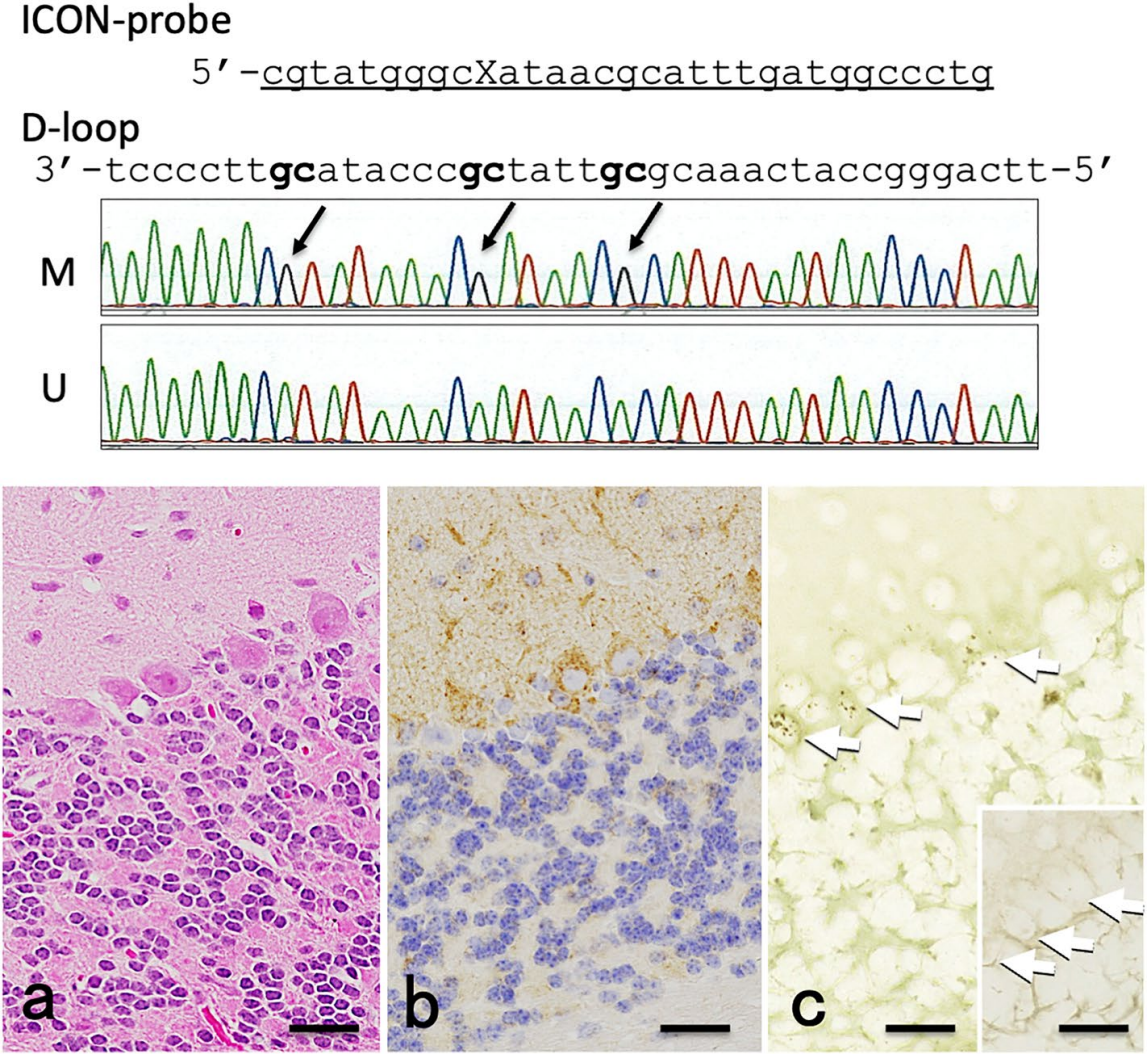
Demonstration of methylated cytosine in mitochondrial DNA. The* upper panel* shows the ICON sequence complementary to the corresponding mitochondrial D-loop area rich in CpG sites (D-loop,* bold face*). Bisulfite sequencing of mouse brain tissue sample demonstrates that 25% of colonies show methylation at CpG sites in the mitochondrial D-loop (*middle panel*,* arrows*, M), while the rest show an unmethylated pattern (*middle panel*, U). In mouse cerebellum, (*lower panel*,** a**, HE), mitochondrial localization by anti-MTCO-1 antibody appears, albeit tends to be abundant in the Purkinje cells, rather ubiquitous mitochondria distribution (*lower panel*,** b**, MT-1). By ICON histochemistry, accumulation of mitochondrial DNA methylation is observed in Purkinje cells in the cerebellum (*lower panel*,** c**,* white arrows*). No significant signals are observed by negative control (*lower panel*,** c**,* right lower insert*,* white arrows*). Each* scale bar* indicates 50 µm

By bisulfite sequencing, three of 12 colonies in mouse brain tissue showed methylation at CpG sites in mitochondrial D-loop (Fig. 4 upper panel, arrows, M), while the rest showed an unmethylated pattern (Fig. 4, upper panel, U). In the mouse cerebellum, (Fig. 4a, HE), mitochondrial localization examined by immunohistochemistry using an MT-1 antibody, showed that, albeit they tended to be abundant in the Purkinje cells and molecular cell layers of the cerebellar cortex, mitochondria were present in virtually all cell types, including the granular layer (Fig. 4b, MT-1). By ICON histochemistry, accumulation of mitochondrial DNA methylation was observed in Purkinje cells in the cerebellum (Fig. 4c, white arrows). No significant signals were observed by negative control (Fig. 4c, right lower insert, white arrows).

## Discussion

Epigenetics, especially DNA methylation, should be analyzed on a cell-by-cell basis because cells may show an epigenetic pattern different from the original cell when they divide to form daughter cells (Kitazawa et al. 2022). Therefore, genome analysis using a mix of multiple cell types may not always correctly reflect the characteristics of the target cell (Kitazawa et al. 2018). On the other hand, there are technical limitations in obtaining epigenomic information from a single cell collected by microdissection (Raine et al. 2022). Therefore, it is very important to conduct “morphology-based epigenetics research” (Kitazawa et al. 2022), in which the region-specific DNA methylation status is observed while maintaining tissue architecture. Since the function of cytosine methylation differs greatly between CpG-islands located near the gene promoter, exons, and introns, the information obtained by simply observing the presence or absence of methylated cytosine by immunohistochemical techniques, with the use of an anti-methylated cytosine antibody, is limited (Frediani et al. 1986). Recently, a tissue-enzyme-chemistry method using methylation-sensitive and -insensitive enzymes has also been developed for the histo-endonuclease-linked detection of methylation sites of the DNA (HELMET) method (Koji et al. 2008). By this HELMET method, methylation dynamics of DNA in germlines is measured on a cell-by-cell basis by digesting the DNA sequence CCGG with methylation-sensitive and methylation-resistant restriction enzymes, while detecting differences in cleavage patterns on tissue sections (Koji et al. 2008). Nevertheless, even with this advanced method, it is not possible to detect DNA methylation specific to sequences involved in specific gene expression. In case of targeting specific gene expression, however, sequence-specific analyses of the methylation status of cytosine on a tissue section are requisite. It is also possible to detect the presence of sequence-specific methylated cytosines by applying a methylation-specific PCR method to methylated and unmethylated cytosines in tissue sections after their treatment with sodium bisulfite (Kitazawa et al. 2018). We have previously demonstrated that this sodium bisulfite-mediated technique shows the distribution of methylation near the RANK gene transcription start site in mouse testis tissue in a sequence-specific manner with the use of a padlock probe specific to the converted base sequence on the tissue section (Kitazawa et al. 2018). Nonetheless, this method has limited applicability because the conversion of cytosine by sodium bisulfite may not be complete due to the strong DNA–protein cross-linking caused by formalin fixation, and it is sometimes difficult to ensure the specificity of probes that recognize DNA sequences after bisulfite conversion. More recently, a single-cell epigenetic visualization assay (EVA) (Kint et al. 2021) has been introduced by combining exonuclease and an antibody bound to the epigenetic mark of interest. Although this is an excellent method for visualizing epigenetic changes in a single cell, it may require the addition of more reaction steps for sensitive visualization of methylated cytosine at a single location, but has not yet been applied to detection in paraffin sections (Kint et al. 2021).

**Fig. 5:**
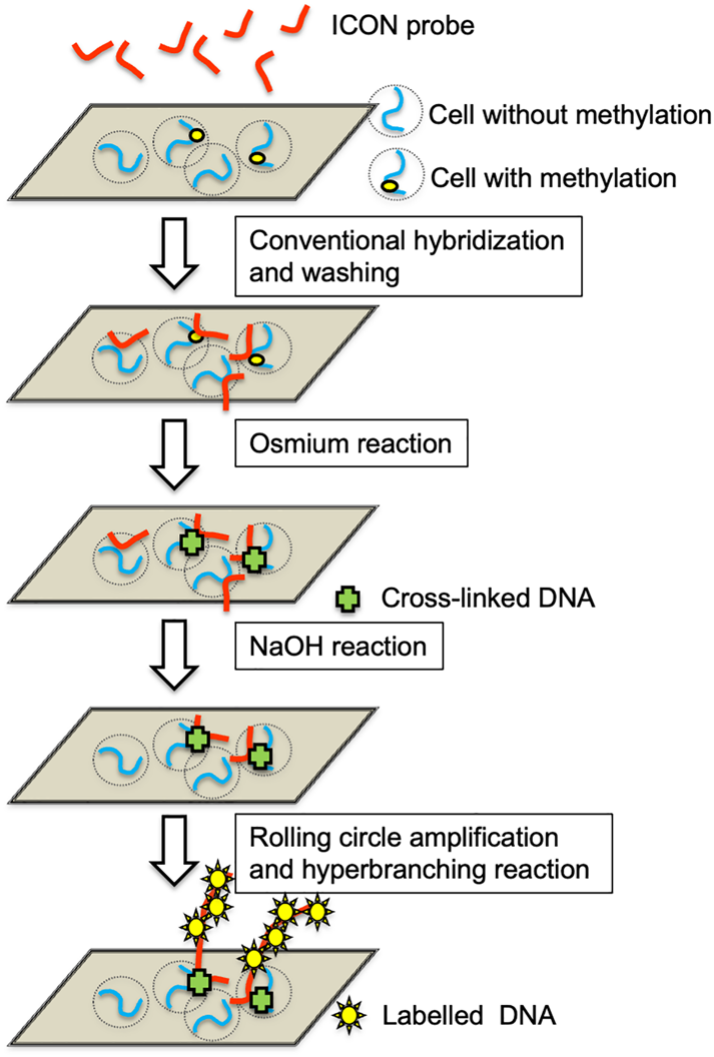
Illustrative histochemical procedure for sequence-specific demonstration of single methylated cytosine on tissue section. After proteinase treatment and denaturation, tissue sections are hybridized with the ICON probe and washed as conventional histo-in situ hybridization with the use of the oligo-DNA probe. By osmium treatment, the ICON probe forms a tight cross-link against methylated cytosine, which remained after the NaOH renaturation process. Padlock probe hybridization and the subsequent rolling-circle amplification elongates the 3’-tail of the hybridized ICON probe with repeated sequences. The hyperbranching reaction by the biotin-labeled probe amplifies this reaction and facilitates signal detection under a light microscope. Because the ICON probe is strongly cross-linked to the nuclear DNA of the target cell, subsequent elongation and multiplication reactions proceed like a tree growing in soil with its roots firmly planted, enabling the demonstration of a single methylated cytosine in situ

In this study, we have developed a method for in situ detection of the methylated state of cytosine at the single nucleotide level, specifically for the base sequence by the use of a probe that forms a complex with high affinity for methylated cytosine. The probe is termed an ICON probe (Okamoto and Tainaka 2005), and is custom-synthesized by GeneDesign, Inc. After forming a molecular hybrid with the target complementary DNA, the ICON probe forms a strong cross-link by complex formation with the target DNA when cytosine is methylated by treatment with a reaction reagent containing potassium osmate (Okamoto and Tainaka 2005). Using this property, a method for visualizing the presence of methylated cytosine in a specific sequence in a chromosome by fluorescent in situ hybridization (MeFISH) has already been described (Shiura et al. 2014; Li et al. 2013), in which a labelled ICON probe has been used directly to visualize repeated DNA sequences at a chromosome or nuclear level. In our current study, as shown in Fig. 1, when creating an ICON probe, a region not involved in molecular hybrids (intermediate bending region of about 12–15 bases followed by padlock probe recognition sequence of about 30 bases) is added to the tail part on the 3' side. With a padlock probe that recognizes the tail part, rolling-circle amplification elongates the 3’ tail of the hybridized ICON probe with the repeated sequence. Following the hyperbranching reaction coupled with biotin labeling, the sensitivity of visualizing the methylation of one base in situ, compared with isotopic labeling, is achieved. Indeed, in the current study, when using purified DNA samples on nylon membranes (Fig. 2a) or on glass slides (Fig. 2b), the methylated and unmethylated states were clearly distinguishable under strong reproving conditions by boiling after the ICON probe was hybridized with the DNA sequence of interest and complexed with potassium osmate treatment, as previously described (Okamoto and Tainaka 2005). In contrast to the use of purified DNA, however, detection of the methylation status in tissues and cells requires accounting for the influence of surrounding nucleoproteins and other factors, as well as the state of fixation. In the present study, examining numerous reaction conditions to determine fairly strict-condition settings was requisite because nonspecific reactions occur even at the cultured-cell level. In particular, the potassium-osmate reaction stage is critical, as has been reported in previous studies (Shiura et al. 2014; Li et al. 2013), in that the authors omitted the ferrate solution and used only potassium osmate to reduce nonspecific binding. After getting the apparent difference by spot hybridization study by the use of radiolabeled-probe (Fig. 2a), with confidence, we searched for the optimal condition for the following hybridization study. In our study, the concentration of potassium osmate treatment was lowered and the duration of the reaction shortened to ensure specificity. Furthermore, when stripping the unbound ICON probe, NaOH was used to prevent the ICON probe from rehybridization. Condition-setting started at the stage of whether it was possible to distinguish between cells that were definitely methylation-positive and those that were negative in a specific region of the gene. The entire procedure is shown as a schema in Fig. 5.

Next, our study was extended to formalin-fixed and paraffin-embedded specimens. The mitochondrial DNA in mouse brain tissue was examined to determine (1) whether DNA methylation is present and (2) in which cells it is distributed. From paraffin sections, and the use of PCR-mediated amplification from bisulfite converted DNA, a fraction of amplified DNA was identified as indeed containing methylation at CpG sites (black arrows in Fig. 4, bisulfite mapping data marked M) at the D-loop of the mitochondria DNA. Although this sodium bisulfite mapping method clearly showed that methylation was present in mitochondrial DNA, it did not provide information on which cells mitochondria were methylated. The ICON method targeting DNA methylation in the D-loop region (Fig. 4 upper panel) showed that most of the signal was localized in Purkinje cells in the cerebellum (Fig. 4, lower panel c).

Immunohistochemistry using specific antibodies for proteins and in situ hybridization using complementary bases for RNA and DNA have been developed and have greatly contributed to the advancement of morphology-based research. It has been challenging, however, to develop an efficient histochemical technique for epigenetics targeting DNA methylation, albeit it being an important issue in the post-genomic era.

In conclusion, our method of combining ICON and rolling-circle amplification is the first that visualizes the presence of a single methylated cytosine in a sequence-specific manner on paraffin sections, and is expected to be applicable to a wide range of future studies. The merit of this method is that the ICON probe is cross-linked to the nuclear DNA of the target cell at first, and the subsequent elongation and multiplication reactions proceed like a tree growing in soil with its roots firmly planted, so the presence of methylated cytosine can be confirmed in situ.

## Supplementary Information

Below is the link to the electronic supplementary material.

Supplementary file1 (DOCX 13 KB)

Supplementary file2 (TIFF 6775 KB)

## Data Availability

All data and materials are available upon request.
