# Supplementary material for: In situ sequence-specific visualization of single methylated cytosine on tissue sections using ICON probe and rolling-circle amplification
Source: Histochem Cell Biol. 2022 Nov 23;159(3):263–73. doi: 10.1007/s00418-022-02165-2 (PMC10006048; doi:10.1007/s00418-022-02165-2)
Supplement: Supplementary file 1 — Supplementary file1 (DOCX 13 KB) [file 418_2022_2165_MOESM1_ESM.docx]

SUPPLEMENTARY DATA

A set of an additional new ICON probe (5’-gcggcXccgccgggcgcgcggggcctagtgagtcgtat-3’) targeting upstream methylated cytosine (35 bases upstream of the target methylated cytosine shown in Figure 1, upper panel indicated by black arrow) with a different padlock probe ligation site and different detecting probes was used. For multicolor metric immunofluorescence staining on glass slides, detected probes were either labeled with green Alexa Fluor 488 or red Alexa Fluor 594 fluorescent agent for differential demonstration of the murine RANK gene target sequences. A glass slide was spotted “1” as a previous target sequence with methylated cytosine, “4” as a new target sequence with methylated cytosine, “3” as a mixture of two target sequences, and “2” as a mixture of non-methylated target sequences (negative control). On glass slides, two kinds of ICON probes differentially identified target DNA showing double methylated DNA as yellow fluorescence (white arrow).
