# Supplementary figures and images for: In situ sequence-specific visualization of single methylated cytosine on tissue sections using ICON probe and rolling-circle amplification
Source: Histochem Cell Biol. 2022 Nov 23;159(3):263–73. doi: 10.1007/s00418-022-02165-2 (PMC10006048; doi:10.1007/s00418-022-02165-2)

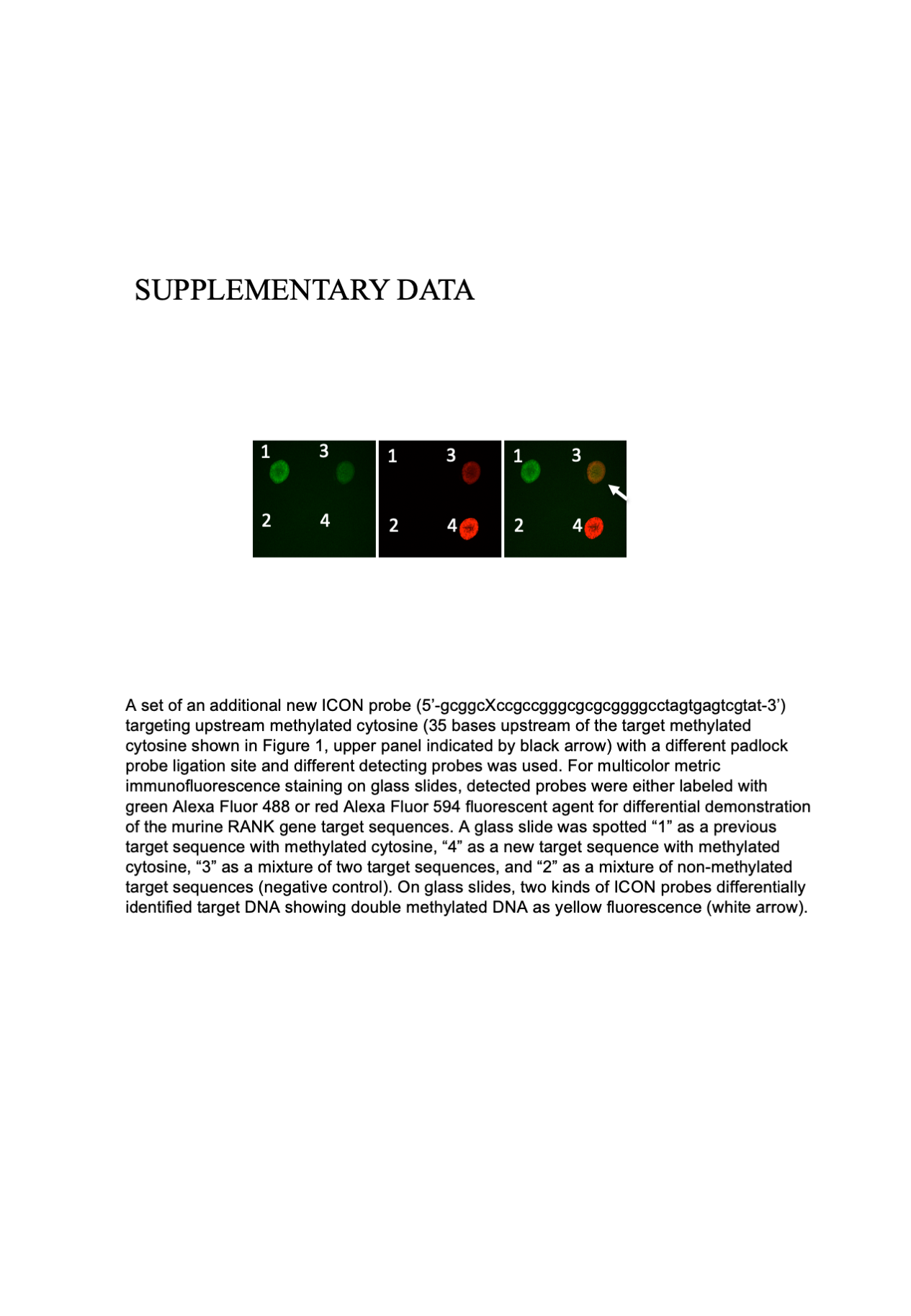

Supplement: Supplementary file 2 — Supplementary file2 (TIFF 6775 KB) [file 418_2022_2165_MOESM2_ESM.tiff]
